# Management of febrile children under five years in hospitals and health centres of rural Ghana

**DOI:** 10.1186/1475-2875-13-261

**Published:** 2014-07-09

**Authors:** Jayne Webster, Frank Baiden, Justina Bawah, Jane Bruce, Mathilda Tivura, Rupert Delmini, Seeba Amenga-Etego, Daniel Chandramohan, Seth Owusu-Agyei

**Affiliations:** 1 Disease Control Department, London School of Hygiene and Tropical Medicine, London, UK; 2 Kintampo Health Research Centre, Kintampo, Ghana

## Abstract

**Background:**

The case management of febrile children in hospitals’ and health centres’ pre-roll out of the new WHO policy on parasitological diagnosis was assessed. The delivery of artemisinin combination therapy (ACT) at these two levels of the health system was compared.

**Methods:**

Structured observations and exit interviews of 1,222 febrile children attending five hospitals and 861 attending ten health centres were conducted in six districts of the Brong Ahafo Region of Ghana. Effectiveness of delivery of case management of malaria was assessed. Proportions of children receiving ACT, anti-malarial monotherapy and antibiotics were described. Predictors of: a febrile child being given an ACT, a febrile child being given an antibiotic and of carers knowing how to correctly administer the ACT were assessed using logistic regression models stratified by hospitals and health centres.

**Results:**

The system’s effectiveness of delivering an ACT to febrile children diagnosed with malaria (parasitologically or clinically) was 31.4 and 42.4% in hospitals and health centres, respectively. The most ineffective process was that of ensuring that carers knew how to correctly administer the ACT. Overall 278 children who were not given an ACT were treated with anti-malarial monotherapy other than quinine. The majority of these children, 232/278 were given amodiaquine, 139 of these were children attending hospitals and 93 attending health centres. The cadre of health staff conducting consultation was a common predictor of the outcomes of interest. Presenting symptoms and examinations conducted were predictive of being given an ACT in hospitals and antibiotic in hospitals and health centres but not of being given an ACT in health centres. Treatment-seeking factors were predictive of being given an ACT if it was more than seven days since the fever began and an antibiotic in hospitals but not in health centres.

**Conclusion:**

Interventions to improve adherence to negative parasitological tests are needed, together with guidance on dispensing of antibiotics, but improving the education of carers on how to administer ACT will lead to the greatest immediate increase in the effectiveness of case management. Guidance is needed on implementation of the new test-based treatment for malaria policy in health facilities.

## Background

After several decades of presumptive treatment of febrile children for malaria in moderate to high transmission areas, a new global policy was introduced in 2010 for the parasitological diagnosis of malaria [1]. The confirmation of diagnosis of malaria must be implemented within the broader diagnosis and treatment of febrile illness as part of the Integrated Management of Childhood Illness (IMCI) [2-4]. The IMCI diagnostic algorithm provides a step-by-step approach to the clinical assessment of a febrile child based on danger signs, symptoms and clinical examinations. The IMCI clinical assessment algorithm has however been poorly implemented in many countries [5-7].

Once a febrile child has been diagnosed, they must be given appropriate treatment for the condition diagnosed and the carer should be given clear instructions on administration of the treatment. Currently the treatment for malaria defined by the national guidelines in sub-Saharan Africa is an artemisinin combination therapy (ACT). All commonly used artemisinin-based combinations, including artesunate-amodiaquine (ASAQ) and artemether lumefantrine (AL), even where the first dose of the treatment is given under observation within the health facility, require further doses to be taken at home. Effective treatment therefore requires that the carer understands, remembers and adheres to instructions on how the treatment should be administered to the child.

Whilst the increased access to rapid diagnostic tests (RDTs) has improved the diagnosis of malaria, the differential diagnoses of other childhood febrile illnesses have not advanced. These depend on robust assessment and interpretation of clinical symptoms and signs and therefore rely on the skills of the clinician. However the clinical assessment of febrile children is often incomplete [8]. This lack of reliable differential diagnoses of febrile illnesses has prompted the concern that the use of RDTs for malaria, whilst decreasing the over-administration of anti-malarials, would result in increased use of antibiotics in children found to be negative for malaria parasites [9]. This has been shown to be the case in several study settings [10-14].

In this study, the case management of febrile children in hospitals and health centres was compared using a health system’s effectiveness algorithm. This system’s effectiveness analysis measures the cumulative effectiveness of all processes and the effectiveness of individual processes in the delivery of case management of febrile children. Adherence and the predictors of adherence to treatment guidelines where children are presumptively and parasitologically diagnosed for malaria are compared. This study was undertaken pre-roll out of the new policy on parasitological diagnosis of malaria and therefore provides a baseline against which future studies on diagnosis and treatment behaviours may be assessed. However, without an assured supply of RDTs, these findings may also be applicable to the programmatic context for long or short periods of time until product supply management chains and sustained funding can be strengthened.

## Methods

### Ethics approval

The study was approved by the Ethics Review Committees of the Ghana Health Service (GHS), the Kintampo Health Research Centre and the London School of Hygiene and Tropical Medicine. Administrative approval was obtained from the respective district and hospital management teams, health workers and carers of children who gave written consent to be observed and interviewed.

### Study setting

The study was conducted in six out of 19 districts of the Brong Ahafo region of Ghana between May and October 2009. These districts were Kintampo North, Kintampo South, Nkoranza North, Nkoranza South and Tain, purposively selected due to their long-standing relationship with Kintampo Health Research Centre. The study setting, design and procedures have been described elsewhere [8]. Malaria transmission is year round in the six study districts and is the major reason for attendance at outpatient departments (OPD).

District hospitals provide primary clinical care for the population living in the catchment area and act as the first level of referral from health centres. The health centre is the main entry point to the health system for clinical care, though services are also available at the community level through the Community-based Health Planning and Services (CHPS) compounds. Health centres provide basic curative, preventative and reproductive health services.

### Study design and sampling procedures

A cross-sectional health facility survey comprising of a health facility audit, structured observations of case management of febrile children and exit interviews with carers was conducted in five hospitals and ten health centres in 2009. The five study district hospitals were selected, with Kintampo North District Hospital being excluded because another study was running at the time with the same target population. The ten health centres were randomly selected from a comprehensive list of health centres in the six districts using probability proportional to size based on OPD attendance in the previous year.

The sample size estimation was based upon the requirements of the health system’s effectiveness analysis, which includes a number of sequential processes in the treatment of a child diagnosed with malaria, and being diagnosed with malaria, being prescribed an ACT, and being given the correct dose of the ACT. The estimation included two stages. In the first stage an estimated 556 febrile children were required to measure a range of outcomes with a frequency of 50% to achieve a 5% precision with a design effect of 2. In the second stage this sample size was increased to maintain a 5% precision on the evaluable sample for each sequential process, including an estimated 60% of children diagnosed with malaria, 70% prescribed an ACT and 90% given the correct dose.

### Data collection

Consent to conduct the study was obtained from the head of each health facility and a written informed consent was obtained from each staff involved in the management of febrile children. Data on their demographic characteristics, professional experience and training were collected using a structured questionnaire. Carers of young children were approached on entering the health facility by study field workers and a written informed consent was obtained for the carers of children meeting the inclusion criteria (child was under five years of age and had a history of fever in the previous two weeks). A field worker observed each step in the management process of the child in the OPD using a structured checklist to record actions and communications by the health workers and the carer. An exit interview was conducted with the carer when ready to leave the facility, which included direct questions on the events during the visit, observation of drugs received and assessment of the knowledge of the carer on how to administer the drugs on leaving the facility. On completion of the process with the carer of the first child the field worker then approached the next eligible child and their carer to enter the facility and repeated the process.

Health facility audits were undertaken at each of the hospitals and health centres to assess the context within which the case management procedures took place.

### Study definitions

Case management of malaria in Ghana is assessed in this study based on the national guidelines at the time which stated: *“In young children, fever or history of fever in the absence of other causes of fever should be considered malaria and treatment commenced immediately without waiting for laboratory results. This is done in the context of IMCI. Confirmatory testing in this age group is not required, but may be considered where available”*[15].

The health system’s effectiveness algorithm had the following four steps: (1) received a clinical or parasitological diagnosis of malaria; (2) given any anti-malarial; (3) given an ACT or quinine; and, (4) carer knew how to give ACT. A diagnosis of malaria without a laboratory confirmatory test was defined as clinical malaria and those cases that were positive for malaria by RDT or microscopy were defined as parasitological malaria. Any anti-malarial included ASAQ, AL, artesunate sulphadoxine-pyrimethamine, artesunate, artesunate suppositories, chloroquine, amodiaquine, and quinine. ACT included ASAQ and AL both of which are recommended first-line treatments for malaria according to the national guidelines [15]. Quinine was also defined as an effective drug at this stage on the assumption that this was given to children who were considered to have severe malaria. Effective knowledge of the carer was defined as knowing that the ACT (ASAQ or AL) should be given twice per day for three days. The data source for the first of these processes was health facility records, and for the others, exit interviews with carers.

The intermediate processes for the system’s effectiveness algorithm were deemed to be ineffective if less that 80% of children completed the process. Cumulative effectiveness of the system is defined as the successful completion of all three intermediary steps, that is, children diagnosed with malaria leaving the health facility with an ACT or quinine with a carer who knows how to correctly administer the treatment.

### Data management and analysis

Data were double-entered and validated using EpiData version 3.1 and Stata 11.0 was used for data processing and analysis. Analyses accounted for the survey design and clustering within health facilities. Findings were stratified by hospital and health centre. The effectiveness of each of the individual intermediate processes and the overall cumulative effectiveness of three processes of the system were analysed.

Pearson’s design-based F-test was used to assess the significance of the difference in the effectiveness of each process and the cumulative effectiveness for all health facilities combined and then stratified by hospitals, all health centres and health centres with microscopy.

Potential predictors of the three outcomes: a febrile child being given an ACT; a febrile child being given an antibiotic; and, of the carer knowing how to administer the ACT amongst those given one were assessed using a univariate (unadjusted) logistic regression model. Categories of potential predictors included: child characteristics, carer characteristics, health worker characteristics, and health facility processes. Health facility processes included: presenting symptoms, clinical examinations, parasitological tests, treatments given and treatment seeking for the child by the carer. Adjusted Wald tests were used to assess the association between each potential predictor and the three outcomes. Predictors with Odds Ratios (ORs) significant at the 10% level (p-values <0.1) were included in multivariable logistic regression models [16] for the three outcomes in order to determine the independent effect of each predictor adjusted for the effect of others. The multivariable models used backward elimination and predictors were considered significant at the 5% level.

## Results

### Characteristics of children and carers

The case management of 1,122 febrile children attending five hospitals and 861 febrile children attending ten health centres was observed and their carers interviewed on exiting the health facilities. Health providers conducting consultations were mainly medical assistants in both hospitals and health centres, but the proportion of children seen by a doctor was higher in hospitals than in health centres (30.1 versus 1.2%; p = 0.0001) (Table 1). The characteristics of the febrile children and their presenting symptoms did not vary between hospitals and health centres. However, a higher proportion of children attending hospitals had health insurance (95.5 versus 81.7%; p = 0.004) than those attending health centres. The carers bringing the child to the hospital were more likely to have completed secondary education (53.8 versus 43.2%; p = 0.05) and to speak Bono, one of the three major local languages, (84.4 versus 66.7%), than those bringing febrile children to health centres.

**Table 1:** Characteristics of providers, carers and children in hospitals and health centres

**Characteristic**		**Hospitals**			**Health centres**			
		**(N = 1,122)**			**(N = 861)**			
		**n**	**%**	**95% CI**	**n**	**%**	**95% CI**	**p**
PROVIDERS								
Cadre (vital signs and history taking)	Medical assistant	22	2.0	0.06, 6.2	3	0.3	0.07, 1.7	0.05
	Nurse	353	31.5	11.0, 63.0	80	9.3	4.7, 17.7	0.04
	Nurse assistant	742	66.1	34.2, 88.0	698	81.1	57.2, 93.2	0.4
Cadre (consultation)	Doctor	338	30.1	10.8, 60.6	10	1.2	0.2, 7.1	0.0001
	Medical assistant	537	47.9	28.7, 67.7	416	48.3	26.2, 71.1	1.0
	Nurse	146	13.0	5.7, 26.9	164	19.1	11.0, 30.9	0.4
	Nurse assistant	110	9.8	1.6, 42.2	162	18.8	7.3, 40.4	0.5
CARERS								
Sex	Male	62	5.5	2.6, 11.4	38	4.4	3.0, 6.4	0.6
	Female	1,060	94.5	88.6, 97.4	823	95.6	93.6, 97.0	0.6
Age	<18	25	2.2	1.5, 3.3	15	1.7	1.2, 2.5	0.3
	18-30	639	57.0	50.1, 63.5	482	56.0	51.0, 60.8	0.8
	≥31	458	40.8	34.9, 47.1	364	42.3	37.1, 47.7	0.7
Education	None	314	28.0	22.0, 34.9	296	34.4	25.2, 44.9	0.3
	Primary	192	17.1	14.9, 19.5	182	21.4	18.0, 24.6	0.05
	Middle/Secondary	604	53.8	49.1,58.5	372	43.2	34.3,52.6	0.05
	Tertiary	12	1.1	0.6, 1.9	11	1.3	0.7, 2.3	0.7
Language (speak any of 3 major dialects)	Twi/Fanti/Asante	1,021	91.0	86.1, 94.3	757	87.9	79.6, 93.1	0.4
	Bono	947	84.4	76.4, 90.1	574	66.7	44.0, 83.6	0.06
	Dagarti	143	12.8	9.5, 16.9	152	17.7	12.7, 24.1	0.1
Relationship to child	Mother	977	87.1	81.8, 91.0	766	89.0	86.6, 91.0	0.4
CHILD								
Age	<12 months	155	13.81	9.6, 19.5	166	19.3	14.2, 25.7	0.1
	12-23 months	329	29.3	26.8, 32.0	238	27.6	24.3, 31.3	0.4
	≥24 months	638	56.9	51.2, 62.3	457	53.1	48.2, 57.9	0.3
Sex	Male	602	53.9	52.2, 55.5	454	52.8	48.0, 57.6	0.7
Health insurance	Yes	1,071	95.5	90.8, 97.8	703	81.7	68.7, 90.0	0.004
Presenting symptoms	Vomiting	338	30.1	27.2, 33.2	260	30.2	26.5, 34.2	1.0
	Cough	406	36.2	25.9, 48.0	243	28.2	23.4, 33.6	0.2
	Diarrhoea	303	27.0	24.2, 30.0	268	31.1	26.9, 35.8	0.1
	Abdominal pain	213	19.0	15.2, 23.4	147	17.1	13.7, 21.0	0.5

Note – languages were those that were coded as speak ‘very well’ or ‘good’ but not is the response was speak ‘not so well’ or ‘not at all’.

### Health system’s effectiveness

**Figure 1:**
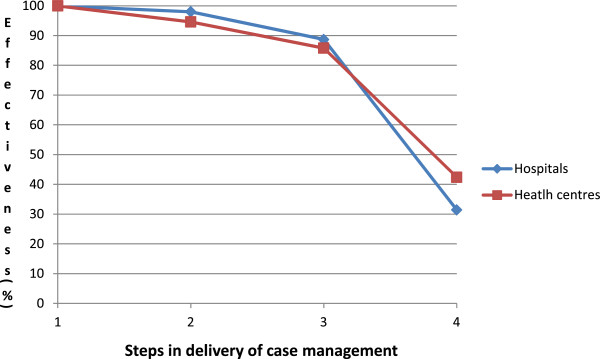
**Health systems effectiveness of case management of malaria in febrile children <5 years.** Note: Step 1 = Receive a clinical diagnosis or are malaria parasite positive (1065 hospitals, 839 health centres); Step 2 = Given any anti-malarial (1044/1065 hospitals, 794/839 health centres); Step 3 = Given an ACT or quinine (945/1044 hospitals, 720/794 health centres); Step 4 = Carer knows how to give ACT [number of days and number of times per day]. (334/888 hospitals, 356/692 health centres).

The majority of febrile children who had a clinical or parasitological diagnosis of malaria were appropriately dispensed either an ACT or quinine (88.7 and 85.8% in hospitals and health centres, respectively) (Figure 1). The most ineffective intermediate process was the carers’ knowledge of how to correctly administer ACT to their children. Only 37.6% of those whose child was given an ACT in hospitals and 51.5% of those given an ACT in health centres knew for how many days and how many times per day they should administer the treatment to the child. This resulted in cumulative health system’s effectiveness of 31.4 and 42.4% in hospitals and health centres, respectively.

### Malaria tests and prescribing of ACT and antibiotics

Only a small proportion (10.2%) of febrile children were tested for malaria parasites in all health facilities; (12.2 and 7.6% in hospitals and health centres, respectively). Whilst all five hospitals had a functioning laboratory and microscopes, this infrastructure was available in only two of the ten health centres. In total, just four RDTs for malaria were conducted in one of the hospitals, although five out of five of the hospitals and nine out of the ten health centres reported having used RDTs previously. Most of the children with a parasitological diagnosis of malaria were given an anti-malarial drug (95% in the hospitals and 98% in the health centres). However, the proportion of children with a parasitological diagnosis of malaria receiving ACT was lower in the hospitals than in health centres (67 versus 93%; p = 0.01) (Table 2). The likelihood of being given an antibiotic did not differ between hospitals and health centres across any of the different diagnosis groups, although numbers of children in parasite test positive and parasite test negative groups were small.

**Table 2:** Diagnosis and treatment with artemisinin combination therapy and antibiotics for febrile children in hospitals and health centres

**Characteristic**			**Hospitals**		**Health centres**			
			**(n = 1122)**		**(n = 861)**			
		**n**	**%**	**95% CI**	**n**	**%**	**95% CI**	**p**
	Had a parasitological test	137	12.2	5.4, 25.3	65	7.6	1.3, 34.0	0.58
Test was positive		82	7.3	3.3, 15.5	44	5.1	1.0, 22.2	0.67
	Given an ACT	55	67.1	54.4, 77.7	41	93.2	77.4, 98.2	0.013
	Given quinine	9	10.8	6.5, 17.5	8	18.2	12.7, 25.4	0.08
	Given an antibiotic	5	6.1	3.2, 11.4	4	9.1	4.0, 19.6	0.38
Test was negative		55	4.9	2.1, 11.1	21	2.4	0.3, 15.4	0.48
	Given an ACT	28	50.9	38.1, 63.6	13	61.9	51.9, 71.0	0.15
	Given quinine	14	25.0	10.8, 47.9	11	52.4	43.5, 61.1	0.03
	Given an antibiotic	11	20.0	6.7, 46.6	8	38.1	27.2, 80.3	0.15
Clinical diagnosis		984	87.8	74.7, 94.6	796	92.5	66.0, 98.7	0.58
	Given an ACT	833	84.7	74.1, 91.5	651	81.8	67.4, 90.7	0.66
	Given quinine	199	20.2	12.3, 31.4	96	12.1	9.5, 15.1	0.06
	Given an antibiotic	275	28.0	12.5, 51.3	302	37.9	27.1, 50.1	0.40

Although more parasite-positive children were given quinine in health centres than in hospitals (10.8 versus 18.2%; p = 0.08) the difference was not statistically significant. Overall, 278 children who were not given an ACT were treated with anti-malarial monotherapy other than quinine. The majority of these children, 232/278, were given amodiaquine; 139 of these were children attending hospitals and 93 attending health centres. A high proportion of the children given such amodiaquine monotherapy were those who were clinically diagnosed (195/232). However, 16 of the 27 children found to be malaria parasite positive in hospitals who were not given an ACT, were given amodiaquine monotherapy.

Collating data from all health facilities, including both hospitals and health centres, febrile children were less likely to be given either an ACT or an antibiotic if they were tested than if they were not tested (68.5 versus 83.4%, p = 0.02 for ACT; 13.8 versus 32.4%, p = 0.007 for antibiotic, respectively) (Table 3). Children accessing hospitals were also less likely to receive an ACT if they were tested compared with not tested (60.9 versus 84.7%; p = 0.006), but this was not the case for children accessing health centres (84.6 versus 81.8%; p = 0.6). In health centres, children tested for malaria were less likely to receive an antibiotic than those who were not tested (18.5 versus 37.9%; p = 0.003) but a similar trend in hospitals was not statistically significant (11.6 versus 28.0%; p = 0.1).

**Table 3:** Treatment received by children tested and not tested for malaria and the result of the test in hospitals and health centres

**Treament given**	**Tested**			**Not Tested**			**MP + ve**			**MP –ve**		
	**N = 203**			**N = 1,780**			**N = 126**			**N = 77**		
	**n**	**%**	**95%CI**	**n**	**%**	**95%CI**	**n**	**%**	**95%CI**	**n**	**%**	**95%CI**
**All HF**												
AM	191	94.1	85.6, 97.7	1,719	96.6	92.3, 98.5						
ACT	139	68.5	53.1, 80.7	1,484	83.4	75.1, 89.4*	121	96.0	82.5, 99.2	70	90.9	66.4, 98.1
AB	28	13.8	8.0, 22.7	577	32.4	20.7, 46.9**	96	76.2	52.2, 90.4	43	55.8	42.0, 68.9*
**Hospitals**												
AM	127	92.0	77.8, 97.4	967	98.3	93.9, 99.5**	78	95.1	59.0, 99.6	49	87.5	49.1, 98.1
ACT	84	60.9	48.2, 72.2	833	84.7	68.6, 93.4**	55	67.1	49.0, 81.2	29	51.8	34.3, 68.9*
AB	16	11.6	3.6, 31.3	275	28.0	8.7, 61.1	5	6.1	2.6, 13.6	11	19.6	3.9, 59.6*
**Health centres**												
AM	64	98.5	80.5, 99.9	752	94.5	85.4, 98.0	43	97.7	15.4, 100	21	100	-
ACT	55	84.6	80.1, 88.3	651	81.8	66.2, 91.2	41	93.2	46.4, 99.5	14	66.7	54.0, 77.3
AB	12	18.5	16.0, 21.3	302	37.9	26.4, 51.0**	4	9.1	1.7, 37.2	8	38.1	25.8, 52.2*

Note: *AM* = anti-malarial; *ACT* = artemisinin combination therapy; *AB* = antibiotic; *HF* = health facilities; *HC* = health centre.

*P = <0.05; **P = <0.01.

Febrile children across all health facilities were more likely to be given an ACT if they were malaria parasite positive than if they were tested and found to be parasite negative (76.2 versus 55.8%; p = 0.03) and less likely to be given an antibiotic (7.1 versus 24.7%; p = 0.01) if parasite positive. When stratifying by hospitals and health centres, the trend was similar but not statistically significant in hospitals and health centres, probably due to low numbers.

### Predictors of receiving an ACT

According to the final model, febrile children taken to a hospital were more likely to receive an ACT if they were ≥12 months of age compared to children <12 months, their carer spoke one of the three major local dialects and if they had already got medicines from a licensed chemical seller (LCS) (adjusted OR 1.34 95% CI 1.14, 1.59; p = 0.002) (Table 4). Febrile children taken to hospitals were less likely to be given an ACT if they presented with a cough (adjusted OR 0.50 95% CI 0.42, 0.60; p < 0.0001), their abdomen was examined (adjusted OR 0.54 95% CI 0.33, 0.89; p = 0.02), it was more than seven days since the fever began (adjusted OR 0.08 95% CI 0.03, 0.22; p = 0.0006) and if and if they had already been given medicines at another health facility (adjusted OR 0.44 95% CI 0.33, 0.60; p = <0.0001).

**Table 4:** Predictors of receiving any artemisinin combination therapy in hospitals and health centres (final models)

**POTENTIAL PREDICTORS**	**n**	**% (95% CI)**	**Unadjusted**		**Adjusted: final model**	
			**OR (95% CI)**	**p**	**OR (95% CI)**	**p**
**1. HOSPITALS**						
**CHILD: Age**						
<12 months	115	74.2 (54.2, 87.5)	1.0	0.01	1.0	0.007
12-23 months	458	81.8 (67.6, 90.6)	1.56 (1.19, 2.05)		1.60 (1.16, 2.20)	
≥24 months	922	83.7 (61.4, 94.3)	1.78 (1.15, 2.76)		1.75 (1.25, 2.46)	
**CARER: Speak any of 3 major dialects**						
No	77	76.2 (54.8,89.5)	1.0	<0.0001	1.0	<0.0001
Yes	841	82.4 (63.1, 92.8)	1.45 (1.28, 1.65)		1.72 (1.47, 2.01)	
**HEALTH WORKER: Cadre (history taking)**						
Nurse	609	82.2 (58.0, 93.9)	1.0	0.09	1.0	0.02
Doctor	17	73.9 (33.3, 94.1)	0.61 (0.16, 2.30)		0.61 (0.20, 1.90)	
Medical assistant	288	81.6 (68.7, 90.0)	0.96 (0.49, 1.89)		1.02 (0.62, 1.69)	
Nurse assistant	3	75.0 (5.9, 99.3)	0.65 (0.04, 10.60)		0.72 (0.04, 14.24)	
**CONSULTATIONS/EXAMINATIONS: Asked about or reported cough**						
No	611	85.5 (68.3, 94.1)	1.0	0.0001	1.0	<0.0001
Yes	306	75.4 (58.0, 87.1)	0.52 (0.40, 0.67)		0.50 (0.42, 0.60)	
**CONSULTATIONS/EXAMINATIONS: Abdomen examined**						
No	803	83.0 (65.3, 92.7)	1.0	0.03	1.0	0.02
Yes	114	74.0 (50.3, 88.9)	0.58 (0.36, 0.94)		0.54 (0.33, 0.89)	
**TREATMENT SEEKING: Time since fever began**						
≤2 days	529	86.3 (64.0,95.7)	1.0	0.0005	1.0	0.0006
3-7 days	370	80.8 (63.4, 91.1)	0.67 (0.38, 1.18)		0.68 (0.39, 1.18)	
>7 days	18	36.0 (9.5, 75.0)	0.09 (0.03, 0.23)		0.08 (0.03, 0.22)	
**TREATMENT SEEKING: Given medicines at other HF**						
No	870	82.8 (65.5, 92.4)	1.0		1.0	0.0001
Yes	47	67.1 (42.4, 85.0)	0.43 (0.31, 0.58)	<0.0001	0.44 (0.33, 0.60)	
**TREATMENT SEEKING: Got medicines at an LCS**						
No	593	79.2 (57.6, 91.4)	1.0	0.0002	1.0	0.002
Yes	324	87.1 (72.9, 94.4)	1.78 (1.39, 2.27)		1.34 (1.14, 1.59)	
**2. HEALTH CENTRES**						
**CARER: Mother of child**						
No	86	90.5 (78.9, 96.1)	1.0	0.0004	1.0	0.0005
Yes	620	80.9 (66.0, 90.3)	0.44 (0.30, 0.65)		0.44 (0.30, 0.65)	
**HEALTH WORKER: Cadre (history taking)**						
Nurse	556	79.7 (63.6, 89.8)	1.0	0.0007	1.0	0.006
Doctor	4	100.0	-		-	
Medical assistant	70	87.5 (76.9, 93.7)	1.79 (1.08, 2.95)		1.84 (1.02, 3.29)	
Nurse assistant	76	96.2 (95.7, 96.7)	6.47 (3.07, 13.6)		6.36 (2.90, 13.95)	
**BLOOD TESTS: Malaria parasite negative**						
No	693	82.5 (67.8, 91.3)	1.0	0.001	1.0	0.02
Yes	13	61.9 (52.9, 70.1)	0.34 (0.20, 0.60)		0.49 (0.27, 0.89)	
**TREATMENTS: Given quinine**						
No	80	70.2 (48.9, 85.3)	1.0	0.01	1.0	0.02
Yes	626	83.8 (69.9, 92.0)	2.20 (1.25, 3.86)		2.09 (1.15, 3.77)	

*LCS* = licensed chemical seller.

In health centres, children were more likely to receive an ACT if the person taking their history and vital signs was a medical assistant or a nurse assistant (medical assistant: adjusted OR 1.84 95% CI 12.02; nurse assistant: adjusted OR 6.36 95% CI 2.90, 13.95; p = 0.006) and if they were given quinine (adjusted OR 2.09 95% CI 1.15, 3.77). Conversely, children attending health centres were less likely to be given an ACT if their carer was their mother (adjusted OR 0.44 95% CI 0.30, 0.65; p = 0.0005) and if they tested negative for malaria parasites (adjusted OR 0.49 95% CI 0.27, 0.89; p = 0.02).

### Predictors of receiving an antibiotic

In the final model, febrile children attending a hospital were more likely to be given an antibiotic if they were seen in consultation by a nurse or health assistant than medical assistant (nurse: adjusted OR2.02 95% CI 1.55, 2.64, health assistant: adjusted OR 5.94 95% CI 3.10, 11.40; p = 0.0001) if they had presented symptoms of diarrhoea (adjusted OR 4.99 95% CI 3.72, 6.68; p = <0.0001) or abdominal pain (adjusted OR 2.31 95% CI 1.33, 4.00; p = 0.006), or sought treatment or drugs elsewhere first (adjusted OR 1.38 95% CI 1.06, 1.79; p = 0.02) (Table 5). Children seeking treatment at hospitals were less likely to be given an antibiotic if their history and vital signs were taken by a doctor rather than a nurse (adjusted OR 0.22 95% CI 0.05, 1.0; p = <0.0001), if they were weighed (adjusted OR 0.14 95% CI 0.07, 0.29; p = <0.0001) or examined for dehydration (adjusted OR 0.09 95% CI 0.02, 0.34; p = 0.002) and if they had a positive malaria test (adjusted OR 0.19 95% CI 0.08, 0.47; p = 0.002).

**Table 5:** Predictors of receiving an antibiotic in hospitals and health centres (final model)

**POTENTIAL PREDICTORS**	**n**	**% (95% CI)**	**Unadjusted**		**Adjusted**	
			**OR (95% CI)**	**p**	**OR (95% CI)**	**p**
**1. HOSPITALS**						
**HEALTH WORKER: Cadre (history taking)**						
Nurse	227	30.6 (7.9, 69.4)	1.0	<0.0001	1.0	<0.0001
Doctor	1	4.3 (0.6, 27.1)	0.10 (0.02, 0.62)		0.22 (0.05, 1.0)	
Medical assistant	62	17.6 (6.4, 40.0)	0.48 (0.12, 1.91)		0.64 (0.33, 1.26)	
Nurse assistant	1	25.0 (2.6, 80.8)	0.76 (0.07, 8.77)		0.56 (0.07, 4.46)	
**HEALTH WORKER: Cadre (consultation)**						
Medical assistant	117	22.4 (10.7, 41.0)	1.0	0.0001	1.0	0.0001
Doctor	45	13.4 (5.9, 27.6)	0.54 (0.20, 1.41)		0.57 (0.24, 1.38)	
Nurse	52	35.9 (16.0, 62.1)	1.94 (3.87, 13.08)		2.02 (1.55, 2.64)	
Health assistant	74	67.3 (33.8, 89.2)	7.12 (3.87, 13.08)		5.94 (3.10, 11.40)	
Other	1	100.0				
**PRESENTING SYMPTOMS: Diarrhoea**						
No	157	19.2 (4.1, 56.9)	1.0	0.001	1.0	<0.0001
Yes	134	44.2 (22.0, 69.0)	3.34 (1.76, 6.39)		4.99 (3.72, 6.68)	
**PRESENTING SYMPTOMS: Abdominal pain**						
No	219	24.1 (8.0, 53.7)	1.0	0.07	1.0	0.006
Yes	72	33.8 (9.3, 71.7)	1.61 (0.97, 2.68)		2.31 (1.33, 4.00)	
**PHYSICAL EXAMINATIONS: Dehydration**						
No	290	26.2 (8.6, 57.4)	1.0	0.03	1.0	0.002
Yes	1	6.7 (0.3, 64.0)	0.20 (0.05, 0.82)		0.09 (0.02, 0.34)	
**PHYSICAL EXAMINATIONS: weighed**						
No	32	76.2 (60.4, 87.0)	1.0		1.0	<0.0001
Yes	259	24.0 (9.1, 50.0)	0.10 (0.06, 0.17)	<0.0001	0.14 (0.07, 0.29)	
**BLOOD TESTS: Malaria test positive**						
No	286	27.5 (8.9, 59.7)	1.0	0.007	1.0	0.002
Yes	5	6.0 (2.9, 12.2)	0.17 (0.05, 0.56)		0.19 (0.08, 0.47)	
**TREATMENT SEEKING: Sought treatment or drugs elsewhere first**						
No	156	24.1 (7.5, 55.5)	1.0	0.04	1.0	0.02
Yes	135	28.5 (9.6, 59.9)	1.26 (1.01, 1.56)		1.38 (1.06, 1.79)	
**2. HEALTH CENTRES**						
**CHILD: Have health insurance**						
No	44	27.9 (20.6, 36.4)	1.0	0.07	1.0	0.02
Yes	270	38.4 (25.7, 53.0)	1.62 (0.96, 2.73)		1.90 (1.11, 3.23)	
**HEALTH WORKER: Cadre (consultation)**						
Medical assistant	142	34.9 (20.6, 52.6)	1.0	0.0001	1.0	<0.0001
Doctor	1	10.0 (10.0, 10.0)	0.21 (0.11, 0.41)		0.10 (0.05, 0.22)	
Nurse	65	39.6 (20.1, 63.2)	1.23 (0.59, 2.56)		1.05 (0.53, 2.09)	
Health assistant	60	37.0 (25.1, 50.9)	1.10 (0.58, 2.06)		1.05 (0.60, 1.85)	
Other	46	39.0 (25.9, 53.9)	1.19 (0.51, 2.78)		1.40 (0.63, 3.12)	
**PRESENTING SYMPTOMS: cough**						
No	200	32.4 (20.8, 46.6)	1.0	0.03	1.0	0.01
Yes	114	46.9 (32.4, 62.0)	1.85 (1.07, 3.20)		2.24 (1.25, 4.00)	
**PRESENTING SYMPTOMS: diarrhoea**						
No	163	27.5 (17.6, 40.3)	1.0	<0.0001	1.0	<0.0001
Yes	151	56.3 (38.0, 73.1)	3.40 (2.28, 5.09)		4.17 (2.62, 6.64)	
**PRESENTING SYMPTOMS: abdominal pain**						
No	248	34.7 (24.2, 47.0)	1.0	0.03	1.0	0.003
Yes	66	44.9 (27.6, 63.5)	1.53 (1.06, 2.22)		1.82 (1.26, 2.61)	
**BLOOD TESTS: Malaria test positive**						
No	310	37.9 (26.7, 50.7)	1.0	0.0003	1.0	0.0002
Yes	4	9.1 (4.3, 18.3)	0.16 (0.07, 0.34)		0.16 (0.07, 0.34)	
**TREATMENTS: Given any monotherapy**						
No	256	33.7 (21.7, 48.3)	1.0	0.04	1.0	0.02
Yes	58	57.4 (37.1, 75.5)	2.56 (1.14, 5.73)		2.57 (1.15, 5.74)	

There were differences between predictors of febrile children receiving an antibiotic at hospitals and health centres. Predictors of receiving an antibiotic in health centres included having health insurance (adjusted OR 1.90 95% CI 1.11, 3.23; p = 0.02), presenting with a cough (adjusted OR2.24 95% CI 1.25, 4.00; p = 0.01), diarrhoea (adjusted OR 4.17 95% CI 2.62, 6.64; p = <0.0001), or abdominal pain (adjusted OR 1.82 95% CI 1.26, 2.62; p = 0.003), and being given any anti-malarial monotherapy (adjusted OR 2.54 95% CI 1.31, 4.92; p = 0.03). Being seen by a doctor in consultation (adjusted OR 0.10 95% CI 0.05, 0.22; p < 0.0001) and having a positive malaria test (adjusted OR 0.26 95% CI 0.16, 0.41; p = 0.0002) were negative predictors of receiving an antibiotic for febrile children attending health centres.

### Predictors of knowing how to give an ACT

In the final model, carers of febrile children taken to hospitals and given an ACT were more likely to know that the child should be given the treatment twice a day for three days if the child was aged 12-23 months compared to if they were <12 months of age (adjusted OR 1.73 95% CI 1.25, 2.39; p = 0.001), and if they were seen in consultation with a nurse or health assistant compared to a medical assistant (nurse: adjusted OR 2.72 95% CI 1.05, 7.02, health assistant: adjusted OR 5.88 95% CI 1.96, 17.6; p = 0.05) (Table 6). Carers were less likely to know how to correctly administer the ACT to their child if they had health insurance (adjusted OR 0.57 95% CI 0.35, 0.92; p = 0.03), the child’s history was taken by a medical assistant compared with a nurse and if they had a malaria test.

**Table 6:** Predictors of knowing how to give an artemisinin combination therapy in hospitals and health centres (final model)

**POTENTIAL PREDICTORS**	**n**	**% (95% CI)**	**Unadjusted**		**Adjusted**	
			**OR (95% CI)**	**p**	**OR (95% CI)**	**p**
**1. HOSPITALS**						
**CHILD: Age**						
<12 months	30	26.1 (3.8, 76.1)	1.0		1.0	0.001
12-23 months	91	33.8 (7.2, 77.1)	1.45 (0.96, 2.18)		1.73 (1.25, 2.39)	
≥24 months	215	40.3 (12.1, 76.9)	1.92 (1.08, 3.39)		2.23 (0.94, 5.33)	
**CHILD: Has health insurance**						
No	24	54.6 (11.0, 92.1)	1.0	0.09	1.0	0.03
Yes	312	35.7 (9.2, 75.3)	0.46 (0.19, 1.14)		0.57 (0.35, 0.92)	
**HEALTH WORKER: Cadre (history taking)**						
Nurse	310	50.9 (19.8, 81.3)	1.0	0.002	1.0	0.002
Doctor	0		-		-	
Medical assistant	25	8.7 (1.5, 37.4)	0.09 (0.02, 0.37)		0.10 (0.04, 0.29)	
Nurse assistant	1	33.3 (0.6, 97.6)	0.48 (0.05, 4.44)		0.60 (0.05, 7.31)	
**HEALTH WORKER: Cadre (consultation)**						
Medical assistant	127	31.4 (8.1, 70.5)	1.0	0.03	1.0	0.05
Doctor	72	25.4 (2.1, 84.2)	0.74 (0.15, 3.64)		1.06 (0.33, 3.50)	
Nurse	56	45.9 (13.6, 82.1)	1.85 (0.61, 5.65)		2.72 (1.05, 7.02)	
Health assistant	80	77.7 (47.0, 93.2)	7.59 (2.36, 24.40)		5.88 (1.96, 17.6)	
Other	1	100	-		-	
**BLOOD TESTS: Has a malaria test**						
No	330	39.6 (11.1, 77.6)	1.0	0.004	1.0	0.009
Yes	6	7.1 (0.8, 43.6)	0.12 (0.03, 0.45)		0.18 (0.05, 0.60)	
**2. HEALTH CENTRES**						
**HEALTH WORKER: Cadre (history taking)**						
Nurse	301	54.1 (25.7, 80.1)	1.0	0.004	1.0	0.006
Doctor	3	75.0 (13.3, 98.3)	2.54 (0.11, 60.14)		7.27 (0.05, >100.0)	
Medical assistant	43	61.4 (18.9, 91.6)	1.35 (0.48, 3.77)		1.45 (0.56, 3.74)	
Nurse assistant	10	13.2 (10.7, 16.1)	0.13 (0.04, 0.40)		0.04 (0.01, 0.17)	
**HEALTH WORKER: Cadre (consultation)**						
Medical assistant	143	42.7 (13.4, 78.2)	1.0	0.0001	1.0	0.006
Doctor	1	12.5 (12.5, 12.5)	0.19 (0.04, 0.83)		0.13 (0.03, 0.58)	
Nurse	83	65.9 (36.8, 86.5)	2.59 (0.91, 7.37)		2.30 (0.82, 6.44)	
Health assistant	102	72.9 (49.8, 87.9)	3.60 (0.78, 16.72)		3.65 (0.80,, 16.7)	
Other	28	28.9 (5.8, 72.9)	0.54 (0.06, 5.29)		5.12 (0.60, 43.7)	
**PRESENTING SYMPTOMS: Lethargy**						
No	9	69.2 (42.7, 87.2)	1.0	0.01	1.0	0.006
Yes	348	50.2 (24.2, 76.1)	0.45 (0.25, 0.80)		0.40 (0.22, 0.73	
**PRESENTING SYMPTOMS: Oedema**						
No	331	49.4 (23.4, 75.7)	1.0	0.02	1.0	0.04
Yes	26	72.2 (53.4, 85.5)	2.66 (1.16, 6.09)		2.67 (1.03, 6.92)	
**BLOOD TEST: Malaria test negative**						
No	356	51.4 (25.1, 76.9)	1.0	0.09	1.0	0.04
Test	1	7.7 (0.3, 70.8)	0.08 (0.01, 1.65)		0.08 (0.01, 0.91)	

Amongst carers who took their febrile children to health centres and were given an ACT, the carer was less likely to have the correct knowledge of ACT administration if the child’s history and vital signs were taken by a nursing assistant in comparison with a nurse (adjusted OR 0.04 95% CI 0.01, 0.17; p = 0.006)), if consultation was conducted by a doctor rather than a medical assistant (adjusted OR 0.13 95% CI 0.03, 0.58; p = 0.006) and if the child had a malaria test which was negative (adjusted OR 0.08 95% CI 0.01, 0.91; p = 0.04).

## Discussion

In this study, the treatment of febrile children with anti-malarials and antibiotics was described based upon clinical and parasitological diagnosis comparing with and contrasting those attending hospitals from those attending health centres.

The study had several limitations. Whilst majority of the data was taken from structured exit interviews with carers of febrile children, some of the data including that of presenting symptoms and examinations conducted were based on structured observations. It is possible that both health workers and carers of children may have changed their behaviours because they were being observed. Such Hawthorne effects are well documented [17,18]. It was assumed that the behaviours observed here were ‘best behaviours’ of both the health workers and the carers. As the cumulative system’s effectiveness was rather low in the hospitals and health centres, any positive change in behaviour due to Hawthorne effect was assumed to be minimal. The enrolment of children and their carers was based upon the number of observations and interviews that can be conducted per day by the available number of field workers and the selection of children were not random. The time of attendance was not, however, a significant predictor in univariate analyses of receiving an ACT, an antibiotic or of the carer knowing how to administer antibiotics and therefore did not impact on the study findings. A stock-take of ACT and antibiotics was not conducted on the day of the survey in each health facility and this may have impacted on the study findings [19]. However, all hospitals and health facilities had ASAQ or AL in stock on the day of the health facility audit and these audits were undertaken immediately prior to the collection of observation and exit interview data. The cadre of health worker conducting both history and vital signs and doing consultation was recorded; however the individual health worker was not. This was a limitation to the inclusion of further health worker characteristics other than cadre, which was a predictor of all three outcomes.

Although having a positive malaria test was associated with receiving an ACT, one-third of children parasite positive in hospitals were not given an ACT and two-thirds of these children with confirmed malaria who were not given an ACT were given amodiaquine monotherapy. One possibility for this practice may be the relative ease of administering a suspension to children as compared to a tablet. Often, even in studies, artesunate-amodiaquine tablets are crushed in sugared syrup to ease administration to children [20] and in the programmatic setting this would require the availability of such a syrup. More than 10% of children given an ACT in health centres were also given quinine and in hospitals this proportion was approaching 20%. Further studies particularly qualitative studies are needed to explain and understand this practice of giving amodiaquine monotherapy in this setting and of giving quinine alongside ACT.

A rationale for the introduction of malaria test-based management of febrile children is that this will reduce over treatment with ACT. In this study setting based on the use of microscopy, this was found to be true. It is also the case that a greater proportion of those found to be positive for malaria parasites were given an ACT than those who were found to be negative. However, there is still considerable effort needed to convince clinicians to adhere to recommendations for negative test results. These behaviours, however, were in response to parasitological testing with microscopy rather than RDTs and children with a negative RDT have been shown to be more likely to be prescribed an anti-malarial treatment than children with a negative microscopy for malaria in some settings [21].

Whilst a greater proportion of malaria parasite-negative than malaria parasite-positive children were given an antibiotic, a smaller proportion of those tested compared to those not tested were administered an antibiotic and therefore overall the use of testing could be said to have reduced the administration of antibiotics in this study. This is, however, within the context of only a small proportion of children being tested and the situation may differ with increases in the proportion of children tested as the proportion of negative tests compared to those not tested increases.

There were few overlaps in predictors of children receiving an ACT or antibiotic and the carers knowing the correct dose schedule of the ACT received in hospitals and health centres. Characteristics of the child and the carer were infrequently predictors of the three outcomes of interest in this study. There were three exceptions to these amongst those attending a hospital which were: 1) that children of 12 months and above were more likely to be given an ACT; 2) children whose carer spoke one of the three local dialects were more likely to be given an ACT; and, 3) the carers of children 23 to 23 months were more likely to know how to give the ACT correctly. It is likely that the diversity of attendees and their ethnic backgrounds is greater at the district hospital than at the health centres, which would go some way to explaining why speaking a local dialect is not a predictor of a child receiving an ACT in a health centre. It does not however explain why in a hospital, a child should be more likely to receive an ACT if the carer speaks the local dialect, except perhaps if the health workers were worried about communicating how the drug should be used. Data to enable analysis by socio-economic indicators was not collected in this study and therefore are not able to determine whether this predictor is confounded by socio-economic status.

Children attending health centres were more likely to receive an antibiotic if they had national health insurance and the carers of children with health insurance were less likely to know how to correctly administer ACT. The proportion of children with health insurance in this study population was very high (89%) overall and was higher for hospitals than health centres, 96 versus 82%. Further studies are needed to understand the influence of the increasing uptake of national health insurance on the prescribing and possible over-administration of antibiotics. It is possible that carers whose children are administered both ACT and antibiotics are less able to remember how to take ACT due to confusion between the two drugs.

The cadre of health worker conducting the consultation was a common predictor of the outcomes of interest in this study; as doctors in this setting are less likely to over-prescribe both ACT and antibiotics than are lower level cadres of health workers but less likely to ensure the understanding of the carer on how to administer the ACT.

Treatment-seeking factors were more influential in the management of febrile children in hospitals than in health centres particularly in terms of previous treatment seeking for the current febrile episode. Children were less likely to be given an ACT if it was more than seven days since their fever began and if they had already been given medicines at another health facility, but more likely to be given an ACT if they had previously taken a medicine from an LCS. This suggests a perception of hospital prescribers that children were likely to have already been given an ACT if they had previously attended another health facility but not if they had got medicine from and LCS.

As assessed using the health system’s effectiveness approach, the most ineffective process in administering an ACT in this study was that of ensuring that the carer of the child knew how to administer the ACT correctly. This approach provides a quantitative comparison of the proportion of children for whom each defined process in delivering effective case management is achieved. The findings were not disaggregated by categories of diagnosis in this system’s analysis where the focus was on the overall picture. Subsequent disaggregation of the findings by diagnosis showed that the aggregated data masked some issues in treatment with amodiaquine and dispensing of both ACT and quinine for a single febrile episode. These two analytical approaches were used effectively to 1) determine the overall quantitative process problems and then, 2) to illuminate more deeply upon treatment based upon diagnosis.

The efficacy of the ACT depends upon adherence to its correct administration and this in turns depends upon the carer’s knowledge of the correct administration. Adherence can be improved by interventions focussing on provider knowledge and behaviour [22]. However, more research using qualitative methodologies is required in this study setting to understand the barriers to carers’ knowledge and how these can be mitigated within the hospital and health centre settings.

Less than 15% of children were tested for malaria parasites in the five district hospitals included in the study, the rest were clinically diagnosed. The behaviour and process changes required for hospitals and their staff to provide a parasitological test to all febrile children is quite different to that in health centres in which microscopy has not been available in the past. In hospitals, health workers make decisions on the need for a malaria test, generally by microscopy. A guideline of all febrile patients to be tested for malaria would require that they changed this practice. This policy is likely to be less effectively implemented by higher level cadres such as doctors who have been more highly trained in using their clinical judgement based upon signs and symptoms. In a study in Western Kenya, the proportion of patients given a diagnostic test post introduction of the new parasitological diagnosis guidelines did not increase, but the majority of these tests were by RDT rather than microscopy [23]. More studies are needed on the programmatic implementation of the new parasitological diagnosis of malaria guidelines in hospitals to understand 1) whether all patients are given a parasitological test; 2) the impact of having a parasitological test on overall quality of care; and, 3) the cost and time (for health staff and patients) implications given the generally high patient loads in district hospitals.

## Conclusion

Interventions are needed to improve case management of febrile children in hospitals and health centres. Non-adherence to malaria microscopy negative results is a problem in both hospitals and health centres with microscopy and prescribing anti-malarial monotherapy for parasite-positive children is not uncommon in hospitals. A high proportion of carers of children did not know how to administer ACT correctly, and this problem is more acute in hospitals than health centres. This suggests that education on drug administration that carers receive in the health facilities is inadequate for its purpose. Predictors of being given an ACT, given an antibiotic and of the carer knowing how to administer the ACT are different in hospitals and health centres and therefore different interventions are need to improve case management of febrile children at these two levels of the health system. Studies to direct appropriate and effective interventions in hospitals are particularly needed.

## Competing interests

The authors have declared that there are no competing interests.

## Authors’ contributions

JW conceived and designed the study, analysed the data and drafted the manuscript. FB conceived and designed the study, implemented the study, and critically reviewed the manuscript. JuB, MT, RD and SA-E implemented the study, managed the data and critically reviewed the manuscript. JaB analysed the data and critically reviewed the manuscript. DC conceived and designed the study, and critically reviewed the manuscript. SO-A conceived and designed the study, implemented the study, and critically reviewed the manuscript. All authors read and approved the final manuscript.

## References

[B1] WHO Guidelines for the Treatment of Malaria 2010 2 Geneva: World Health Organization 25473692

[B2] Gove S Integrated management of childhood illness by outpatient health workers: technical basis and overview. The WHO working group on guidelines for integrated management of the sick child Bull World Health Organ 1997 75 Suppl 1 7 24 9529714 PMC2486995

[B3] WHO/UNICEF Handbook IMCI Integrated Managment of Childhood Illnesses 2005 Geneva: World Health Organization/United Nations Childrens’ Fund

[B4] WHO Guidelines for the Treatment of Malaria 2006 2 Geneva: World Health Organization

[B5] Bryce J Victora CG Habicht JP Vaughan JP Black RE The multi-country evaluation of the integrated management of childhood illness strategy: lessons for the evaluation of public health interventions Am J Public Health 2004 94 406 415 14998804 10.2105/ajph.94.3.406 PMC1448266

[B6] Horwood C Vermaak K Rollins N Haskins L Nkosi P Qazi S An evaluation of the quality of IMCI assessments among IMCI trained health workers in South Africa PLoS One 2009 4 e5937 19536288 10.1371/journal.pone.0005937 PMC2693922

[B7] Rowe AK Onikpo F Lama M Cokou F Deming MS Management of childhood illness at health facilities in Benin: problems and their causes Am J Public Health 2001 91 1625 1635 11574325 10.2105/ajph.91.10.1625 PMC1446844

[B8] Baiden F Owusu-Agyei S Bawah J Bruce J Tivura M Delmini R Gyaase S Amenga-Etego S Chandramohan D Webster J An evaluation of the clinical assessments of under-five febrile children presenting to primary health facilities in rural Ghana PLoS One 2011 6 e28944 22174932 10.1371/journal.pone.0028944 PMC3236777

[B9] Baiden F Webster J Owusu-Agyei S Chandramohan D Would rational use of antibiotics be compromised in the era of test-based management of malaria? Trop Med Int Health 2011 16 142 144 21087379 10.1111/j.1365-3156.2010.02692.x

[B10] Msellem MI Martensson A Rotllant G Bhattarai A Stromberg J Kahigwa E Garcia M Petzold M Olumese P Ali A Bjorkman A Influence of rapid malaria diagnostic tests on treatment and health outcome in fever patients, Zanzibar: a crossover validation study PLoS Med 2009 6 e1000070 19399156 10.1371/journal.pmed.1000070 PMC2667629

[B11] Bastiaens GJ Schaftenaar E Ndaro A Keuter M Bousema T Shekalaghe SA Malaria diagnostic testing and treatment practices in three different *Plasmodium falciparum* transmission settings in Tanzania: before and after a government policy change Malar J 2011 10 76 21457570 10.1186/1475-2875-10-76 PMC3080800

[B12] Batwala V Magnussen P Nuwaha F Antibiotic use among patients with febrile illness in a low malaria endemicity setting in Uganda Malar J 2011 10 377 22183039 10.1186/1475-2875-10-377 PMC3258227

[B13] Nadjm B Amos B Mtove G Ostermann J Chonya S Wangai H Kimera J Msuya W Mtei F Dekker D Malahiyo R Olomi R Crump JA Whitty C Reyburn H WHO guidelines for antimicrobial treatment in children admitted to hospital in an area of intense *Plasmodium falciparum* transmission: prospective study BMJ 2010 340 c1350 20354024 10.1136/bmj.c1350 PMC2847687

[B14] D’Acremont V Kahama-Maro J Swai N Mtasiwa D Genton B Lengeler C Reduction of anti-malarial consumption after rapid diagnostic tests implementation in Dar es Salaam: a before-after and cluster randomized controlled study Malar J 2011 10 107 21529365 10.1186/1475-2875-10-107 PMC3108934

[B15] Ministry of Health Guidelines for Case Management of Malaria in Ghana 2009 Accra: Ministry of Health

[B16] Katz MH Multivariable Analysis: A Practical Guide for Clinicians and Public Health Researchers 2011 Cambridge: Cambridge University Press

[B17] Rowe SY Olewe MA Kleinbaum DG McGowan JE Jr McFarland DA Rochat R Deming MS The influence of observation and setting on community health workers’ practices Int J Qual Health Care 2006 18 299 305 16675475 10.1093/intqhc/mzl009

[B18] Leonard K Masatu MC Outpatient process quality evaluation and the Hawthorne effect Soc Sci Med 2006 63 2330 2340 16887245 10.1016/j.socscimed.2006.06.003

[B19] Hensen B Paintain LS Shretta R Bruce J Jones C Webster J Taking stock: provider prescribing practices in the presence and absence of ACT stock Malar J 2011 10 218 21812948 10.1186/1475-2875-10-218 PMC3163227

[B20] Oyakhirome S Potschke M Schwarz NG Dornemann J Laengin M Salazar CO Lell B Kun JF Kremsner PG Grobusch MP Artesunate–amodiaquine combination therapy for falciparum malaria in young Gabonese children Malar J 2007 6 29 17352806 10.1186/1475-2875-6-29 PMC1831475

[B21] Reyburn H Mbakilwa H Mwangi R Mwerinde O Olomi R Drakeley C Whitty CJ Rapid diagnostic tests compared with malaria microscopy for guiding outpatient treatment of febrile illness in Tanzania: randomised trial BMJ 2007 334 403 17259188 10.1136/bmj.39073.496829.AE PMC1804187

[B22] Yeung S White NJ How do patients use antimalarial drugs? A review of the evidence Trop Med Int Health 2005 10 121 138 15679555 10.1111/j.1365-3156.2004.01364.x

[B23] Skarbinski J Ouma PO Causer LM Kariuki SK Barnwell JW Alaii JA de Oliveira AM Zurovac D Larson BA Snow RW Rowe AK Laserson KF Akhwale WS Slusker L Hamel MJ Effect of malaria rapid diagnostic tests on the management of uncomplicated malaria with artemether-lumefantrine in Kenya: a cluster randomized trial Am J Trop Med Hyg 2009 80 919 926 19478249

